# Identification of Glioma Phenotypic Subtypes From Multimodal MRI Data Using Hierarchical Multi‐Kernel Learning

**DOI:** 10.1002/cam4.71572

**Published:** 2026-02-01

**Authors:** Junyu Yan, Min Hao, Tong Wang, Qi Yang, Congcong Jia, Wenju Niu, Yan Tan, Hui Zhang, Hongyan Cao, Guoqiang Yang

**Affiliations:** ^1^ Department of Radiology First Hospital of Shanxi Medical University Taiyuan China; ^2^ Academy of Medical Sciences Shanxi Medical University Taiyuan China; ^3^ Department of Health Statistics, Shanxi Provincial Key Laboratory of Major Diseases Risk Assessment, School of Public Health Shanxi Medical University Taiyuan China; ^4^ College of Medical Imaging Shanxi Medical University Taiyuan China; ^5^ MOE Key Laboratory of Coal Environmental Pathogenicity and Prevention Shanxi Medical University Taiyuan China; ^6^ Shanxi Key Laboratory of Intelligent Imaging and Nanomedicine First Hospital of Shanxi Medical University Taiyuan China; ^7^ Intelligent Imaging Big Data and Functional Nano‐Imaging Engineering Research Center of Shanxi Province First Hospital of Shanxi Medical University Taiyuan China

**Keywords:** glioma, hierarchical multi‐kernel learning, multimodal, phenotypic subtype, radiomics

## Abstract

**Background:**

Gliomas are the most common primary brain tumors, exhibiting significant phenotypic variability even within the same grade. Identifying glioma subtypes through non‐invasive methods could improve patient management.

**Methods:**

In this study, we applied hierarchical multi‐kernel learning to identify glioma phenotypic subtypes using MRI data (T1CE and T2FLAIR) from the First Hospital of Shanxi Medical University (FHSXMU) and Shanxi Provincial People's Hospital (SPPH) (*n* = 246). We further validated our findings using an independent dataset of similar tumor characteristics from The Cancer Genome Atlas/The Cancer Imaging Archive (TCGA/TCIA) (*n* = 144). Additionally, we analyzed pathway activity across glioma subtypes from the TCGA/TCIA dataset and employed five machine learning models, namely kernel partial least squares with the genetic algorithm (GA‐KPLS), random forest, the least absolute shrinkage and selection operator, K‐Nearest Neighbor, and Naïve Bayes, to predict isocitrate dehydrogenase (IDH) genotype from the FHSXMU/SPPH dataset.

**Results:**

We identified 2 glioma phenotypic subtypes, high‐risk and low‐risk groups. These groups showed significant differences in overall survival (*p* < 0.05) and were associated with distinct signaling pathways. The JAK–STAT and TGF‐β pathways were activated in the high‐risk group, while the Hypoxia and p53 pathways were activated in the low‐risk group. Among the machine learning models, the GA‐KPLS model demonstrated the highest predictive performance for the IDH genotype, achieving an area under the curve of 0.819.

**Conclusion:**

Our study provides a non‐invasive method to identify glioma phenotypic subtypes, reveal distinct signaling pathways, and define therapeutically homogeneous patient subgroups that could guide targeted therapy.

## Graphical abstract

Our study identified glioma phenotypic subtypes based on T1CE and T2FLAIR radiomic features, along with associated pathway activities, providing real‐time insights into tumor dynamics to guide preoperative clinical decision‐making and improve patient management. Additionally, the GA‐KPLS‐based predictive model effectively assessed IDH genotype, enabling preoperative evaluation to inform clinical decisions.

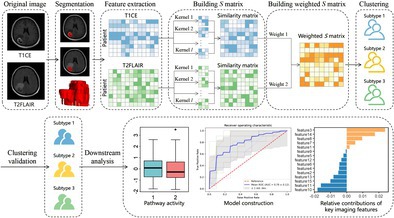

## 1 Introduction

Gliomas, originating from neuroectodermal cells, are the most common primary intracranial tumors of the central nervous system [1]. The five‐year survival probability for patients with gliomas is less than 35% [2]. Patients with identical glioma types and grades frequently exhibit distinct clinical prognoses even if receiving the same surgical resection and chemoradiotherapy treatments [3]. The underlying phenotypic heterogeneity in gliomas may be a key factor contributing to the variability in clinical outcomes and therapeutic responses. Therefore, identifying glioma phenotypic subtypes and detecting the subtype‐specific phenotypic alterations correlated with clinical outcomes is crucial for targeted therapy, as well as for improving prognosis and enabling personalized precision treatment. With the advancement of glioma phenotypic subtyping, magnetic resonance imaging (MRI) has emerged as a non‐invasive diagnostic tool for preoperative assessment of gliomas, offering potential opportunities for risk evaluation in glioma patients [4]. Radiomics improves the accuracy of diagnosis, prognosis, and prediction by performing high‐throughput feature extraction from conventional MRI images of tumor lesions. It captures first‐order, morphological, and textural features, as well as histological information, including image intensity variations and spatial interrelationships, to provide deeper insights into tumor phenotypes and heterogeneity [5, 6, 7].

Unsupervised learning for clustering high‐throughput radiomic features enables more effective phenotypic subtyping of gliomas and enhances the exploration of intrinsic relationships among different subtypes, thereby contributing to a better understanding of tumor phenotypic heterogeneity. Haruka Itakura et al. [8] used consensus clustering on T1 post‐contrast (T1CE) quantitative imaging features from glioblastoma (GBM) patients to identify imaging phenotypes and integrate them with molecular signaling pathways. Gevaert et al. [9] employed hierarchical clustering to combine quantitative imaging features of GBM, including contrast‐enhanced T1CE and T2 fluid‐attenuated inversion recovery image (T2FLAIR), with genomic data. Additionally, Haldar et al. [10] used the K‐means method to cluster pediatric low‐grade gliomas based on BRAF mutation frequency, utilizing T1‐weighted pre‐ (T1) and post‐contrast (T1‐Gd), T2‐weighted, and T2FLAIRsequences.

Single MR sequences have limitations in tumor feature studies, and integrating multimodal images is more meaningful for research. Previous studies combined radiomic features from single or multiple sequences but did not fully account for the precise contribution of each sequence to the feature representation. In this study, we employed an unsupervised multi‐kernel learning method, hierarchical multi‐kernel learning (hMKL) [11], utilizing multi‐kernel learning to effectively integrate information from T1CE and T2FLAIR, enhancing model accuracy and robustness, and enabling precise glioma phenotype classification.

## 2 Materials and Methods

### 2.1 Data

#### 2.1.1 Patients

The retrospective study included patients with histologically confirmed gliomas from our institutions, including the First Hospital of Shanxi Medical University (FHSXMU) and Shanxi Provincial People's Hospital (SPPH), as well as data from the The Cancer Imaging Archive/The Cancer Imaging Archive (TCGA/TCIA) projects.

All 344 patients with pathologically confirmed primary gliomas at FHSXMU/SPPH during the period from 12/2011 to 12/2019 were enrolled and screened. A total of 208 gliomas from the TCGA/TCIA project were collected and screened, including the TCGA‐LGG and TCGA‐GBM datasets. All patients identified in this study met the following inclusion criteria: (1) diagnosis of primary gliomas after surgery; (2) available preoperative three‐week MRI data, consisting of contrast‐enhanced T1‐weighted image (T1CE) and T2 fluid‐attenuated inversion recovery image (T2FLAIR) images; (3) confirmed isocitrate dehydrogenase (IDH) and O6‐methylguanine‐DNA methyltransferase (MGMT) statuses; (4) follow‐up time exceeded 2 years or included an endpoint event (suitable for overall survival analysis). Overall survival was calculated from the postoperative pathological diagnosis to death or the last follow‐up. The exclusion criteria include (1) pathologically confirmed grade 1 glioma according to the 2021 WHO glioma classification criteria; (2) the patient has received targeted treatment (including chemotherapy, radiotherapy, and conservative treatment) before surgery; (3) severe image motion artifacts or poor image quality; (4) no postoperative follow‐up or follow‐up loss. Additionally, patients from the TCGA/TCIA project also met the following criteria: each patient must have corresponding mRNA expression data. Ultimately, 390 patients who met the criteria above (246 from FHSXMU/SPPH and 144 from the TCGA/TCIA dataset) were enrolled in our study.

#### 2.1.2 Gene Expression Data

Gene expression data from the TCGA/TCIA dataset were processed by removing features with more than 30% missing values and applying log_2_ (*x* + 1) transformation to reduce skewness and stabilize variance, resulting in 19,938 mRNAs.

#### 2.1.3 IDH Genotyping, and MGMT Promoter Methylation Testing

For patients from the TCGA/TCIA dataset, IDH mutation and MGMT promoter methylation data were obtained from the Genomic Data Commons Data Portal. IDH1/2 mutation status at our institutions was determined via Sanger sequencing. MGMT promoter methylation status was evaluated using pyrosequencing analysis [12]. Specific details can be found in our previous research [13].

#### 2.1.4 MRI Data Acquisition

Preoperative MRI was performed using a 3.0‐T scanner (Signa HDxt, GE Healthcare, USA) equipped with an 8‐channel array coil. The gradient echo contrast‐enhanced T1‐weighted imaging (T1CE) was acquired with a repetition time (TR) of 195 ms and a time to echo (TE) of 4.76 ms. The T2 fluid‐attenuated inversion recovery (T2FLAIR) sequence was acquired with a TR of 8000 ms, a TE of 95 ms, and a time to inversion of 2000 ms. The imaging slice thickness was 5.0 mm with a slice spacing of 1.5 mm. The field of view was 240 × 240 mm^2^ with a matrix size of 256 × 256. A gadolinium chelate contrast agent with a concentration of approximately 0.1 mmol/kg was used for contrast‐enhanced imaging.

### 2.2 Image Co‐Registration and Tumor ROI Segmentation

**FIGURE 1:**
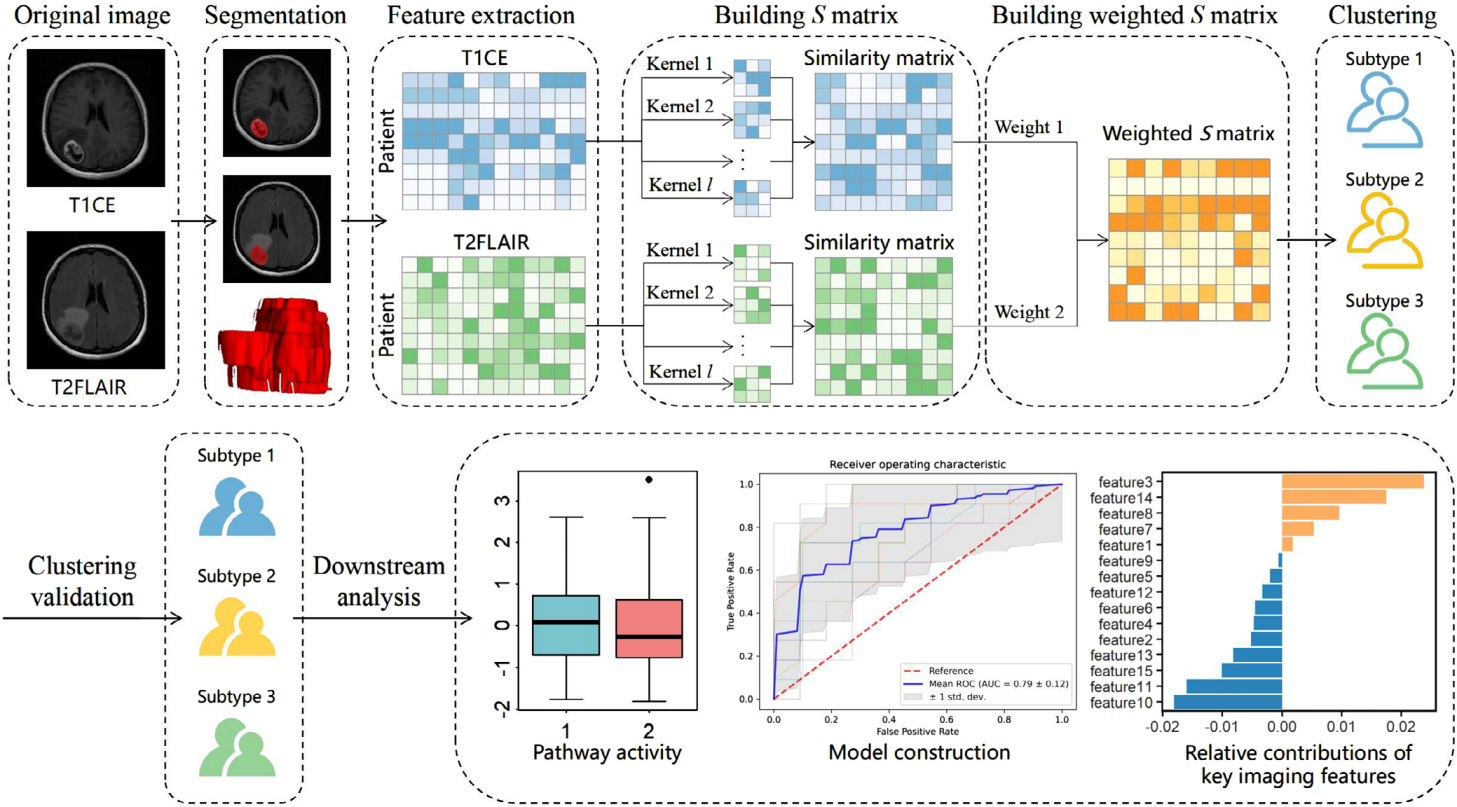
The overview of this study. This study includes tumor segmentation, feature extraction, and unsupervised learning clustering. The flowchart of hMKL begins by calculating the sample‐similarity kernels for each feature matrix (T1CE and T2FLAIR). The Gaussian kernel parameters and weights are optimized under the CIMLR framework, and unsupervised multiple kernel learning (UMKL) is used to optimize fusion weights, resulting in a final similarity matrix. K‐means clustering is then applied to the fused matrix for subtype identification.

The research design process is illustrated in Figure 1. The T2FLAIR images were co‐registered with the corresponding T1CE images using the Oxford Functional MRI Center for the Brain (FMRIB) Linear Image Registration Tool (FLIRT) from the FMRIB Software Library. Two radiologists with extensive experience in brain MRI diagnostic imaging used ITK‐SNAP (http://www.itksnap.org, Version 3.8.0) to convert the DICOM format to NIFTI format and then outlined the tumor region of interest (ROI) slice‐by‐slice on the axial MRI images. Since all images were pre‐registered, the ROIs outlined on the T1CE could be synchronized with the T2FLAIR sequence. Finally, we obtained the volume of interest (VOI) for the tumor. The two physicians were blinded to the patient's pathological grade, clinical information, and other imaging results.

### 2.3 Radiomic Feature Extraction and Selection

Based on the pyradiomics module algorithm provided by FAE (Version 0.5.6) [14], we extracted radiomic features from each sequence of VOIs. Both T1CE and T2FLAIR sequences were extracted with 107 basic radiomic features, including first‐order histogram features (*n* = 18), shape and size features (*n* = 14), and textural features (*n* = 75). Textural features comprise the Gray Level Co‐occurrence Matrix (*n* = 24), Gray Level Dependence Matrix (*n* = 14), Gray Level Run‐Length Matrix (*n* = 16), Gray Level Size Zone Matrix (*n* = 16), and Neighboring Gray Tone Difference Matrix (*n* = 5). At the same time, six image filters, including wavelet transform, square, square root, logarithmic, exponential, and gradient, were used to preprocess the original images and extract the higher‐order radiomic features (*n* = 1579). Each patient's MRI produced a total of 3372 features from the two sequences.

To mitigate dimensionality catastrophe and enhance the understanding of features, we employed the Coefficient of Variation (CV) to perform “filter‐based” feature selection for the T1CE and T2FLAIR sequences. Specifically, we calculated the CV of each feature by determining the ratio of its standard deviation to its mean and converting it into a percentage. Features with less than 30% variation were removed, leaving only those with higher variation. The formula is expressed as $c_{v}=\frac{\sigma }{\mu }$, where σ is the standard deviation and μ is the mean value.

### 2.4 Hierarchical Multi‐Kernel Learning (hMKL)

hMKL [11] was employed to perform kernel fusion on radiomic feature matrices derived from T1CE and T2FLAIR. In Stage 1, the Gaussian kernel parameters and weights for each omics type were optimized under the framework of Cancer Integration via Multikernel Learning (CIMLR) [15]. In Stage 2, the final weighted similarity matrix was obtained using UMKL by optimizing the weights [16]. Based on the fused kernel, K‐means clustering was performed to achieve unsupervised clustering. Detailed formulas and explanations for the above process are provided in Part 1 of Appendix S1.

### 2.5 Estimating the Optimal Number of Clusters

Since we used an unsupervised learning approach without predefined labels, the optimal number of clusters k was estimated by maximizing the sum of the Clustering Prediction Index (CPI) and the Gap statistic [17]. CPI was calculated as the average within‐cluster sum of squares across test samples, while the Gap statistic was determined by comparing the observed within‐cluster sum of squares with its expected value under a null reference distribution [18, 19]. Detailed formulas and explanations for the above methods are provided in Part 2 of Appendix S1. Additionally, the Davies‐Bouldin index (DBI) [20] and Calinski‐Harabasz (CH) index [21] were employed to further validate the clustering results; the specific methodology and results are provided in Part 3 of Appendix S1.

### 2.6 Validation of Clustering Effectiveness

To validate the robustness and reproducibility of our subtype clustering in another dataset, we used the “group_predict” function in the *R* package “SNFtool,” which applies the Similarity Network Fusion (SNF) [22] method for classification. By merging similarity matrices from multiple data modalities or sources into a unified network, it classifies new data samples. Specifically, we first conducted differential radiomic features across subtypes for T1CE and T2FLAIR sequences in the FHSXMU/SPPH dataset using the Kruskal–Wallis *H*‐test, followed by a false discovery rate (FDR) adjustment (FDR < 0.05). This resulted in 1042 T1CE features and 1019 T2FLAIR features. We then matched the features from the TCGA/TCIA dataset with those from FHSXMU/SPPH to ensure data consistency, and then we used the FHSXMU/SPPH dataset as the training set and the TCGA/TCIA dataset as the test set to predict subgroup labels for TCGA/TCIA.

### 2.7 Downstream Statistical Analysis After Subtyping

#### 2.7.1 Pathway and Gene Functional Enrichment Analysis

We used the *R* package “decoupleR” [23] to identify variations in 14 signaling pathway activities across different subtypes of gene expression data. We utilized the top 500 human response genes, ranked by *p*‐value from the PROGENy database, and applied a Multivariate Linear Model to each sample in the TCGA/TCIA data. This model predicted observed gene expression based on the interaction weights of pathways and genes. After fitting the model, the resulting slope t‐values were used as pathway scores. A positive t‐value indicates pathway activation, whereas a negative t‐value suggests pathway inhibition.

We performed Kyoto Encyclopedia of Genes and Genomes (KEGG) [24] analysis on the genes in the key modules using the online tool KOBAS 3.0 [25] to identify overrepresented genes or proteins.

#### 2.7.2 Model Construction

By comparing the differences across subtypes, we constructed predictive models for genotypes that showed significant differences in both datasets. To reduce irrelevant and redundant information, we performed stability feature selection based on penalized logistic regression [26]. Each of the two types of radiomic features, after the Kruskal‐Wallis *H*‐test, was randomly divided into training and testing datasets with an 80:20 split ratio. We then performed penalized logistic regression on the training data using the *R* package “glmnet” for feature selection, recording the results from 100 random splits. The feature selection results were ranked by their selection frequency across these splits, and features selected in ≥ 10% of replicates were chosen as final candidates for further analysis.

We employed five machine learning models, including kernel partial least squares with the genetic algorithm (GA‐KPLS) [27, 28], random forest (RF) [29], the least absolute shrinkage and selection operator (LASSO) [30], K‐Nearest Neighbor (KNN) [31], and Naïve Bayes (NB) [32]. KPLS maps the low‐dimensional input space to a high‐dimensional feature space through the application of kernel methods, enabling samples that are not separable in low‐dimensional space to become linearly separable in the feature space [27]. GA is an optimization algorithm based on the “survival of the fittest” genetic mechanism. In this study, we first used a Gaussian kernel function to construct a kernel matrix for the radiomic feature matrix and then used GA to optimize the parameters of the Gaussian kernel function, including the kernel bandwidth *σ*. The original data was divided into two non‐overlapping datasets: the training dataset and the testing dataset. Through stratified sampling, 80% of the data was used to build the predictive model, while the remaining 20% was used to evaluate the model's predictive performance. For LASSO, the optimal tuning parameter *λ* was chosen by tenfold cross‐validation over a grid of 100 *λ* values, implemented using the *R* package “glmnet.” For RF, KNN, and NB, all parameters were set to their default values, utilizing the *R* packages “randomForest,” “class” and “e1071,” respectively.

The model performances were evaluated using multiple criteria, including the area under the Receiver operating characteristic (ROC) curve (area under the curve, AUC), sensitivity (Se), specificity (Sp), accuracy (ACC), Youden index, F‐measure, G‐means, and Matthews Correlation Coefficient (MCC). The MCC and AUC were primarily used to assess model performance due to their comprehensive evaluation criteria. To compare the performance of GA‐KPLS with four other models (RF, LASSO, KNN, and NB), we applied one‐way ANOVA followed by Dunnett's multiple‐comparison test. *p* < 0.05 was considered statistically significant. The sampling and modeling process was repeated for 1000 iterations in the study to ensure the stability and reliability of the predictions for each model.

#### 2.7.3 Differential and Relative Contributions of Key Imaging Features

Based on the radiomic features after the Kruskal‐Wallis *H*‐test, we performed an enrichment analysis to assess the relative contribution of each feature to the cluster using the hypergeometric test, with an FDR‐adjusted *p*‐value < 0.05. To identify significantly expressed radiomic features, we considered a feature to be over‐ or under‐expressed if its standardized value was larger than the 75th percentile or lower than the 25th percentile. Additionally, we applied more stringent criteria by selecting features that were altered in at least two‐thirds of the samples within a cluster and fewer than one‐third of the samples in at least one other cluster.

## 3 Results

### 3.1 Two MRI Glioma Subtypes Exist

MRI data were obtained from 390 glioma patients, split into two different cohorts: the FHSXMU/SPPH data for subtype identification and the TCGA/TCIA data for validation. Table 1 summarizes the baseline characteristics of the two datasets.

**TABLE 1:** Clinical and molecular characteristics of the FHSXMU/SPPH and TCGA/TCIA datasets.

Characteristics	FHSXMU/SPPH training data (*N* = 246)	TCGA/TCIA test data (*N* = 144)
Overall survival (day)	655.83 ± 472.77	734.94 ± 544.12
Age (year)	48.82 ± 15.12	47.53 ± 14.42
Gender		
Female	136 (55.28)	72 (50.00)
Male	110 (44.72)	72 (50.00)
Grade		
2	82 (33.33)	56 (38.89)
3	78 (31.71)	67 (46.53)
4	86 (34.96)	21 (14.58)
Tumor volume (cm^3^)	42.05 ± 34.33	43.03 ± 50.44
IDH genotype		
Wild type	130 (52.85)	42 (29.17)
Mutant type	116 (47.15)	102 (70.83)
MGMT promoter genotype		
Unmethylation	84 (34.15)	30 (20.83)
Methylation	162 (65.85)	114 (79.17)

*Note:* FHSXMU/SPPH dataset, consisting of 246 patients. TCGA/TCIA dataset, consisting of 144 patients. Overall survival, age, and tumor volume are expressed as *mean* ± *standard* deviation (*x* ± *s*); gender, grade, IDH genotype, and MGMT promoter genotype are tabulated as number (*n*) and percentage.

**FIGURE 2:**
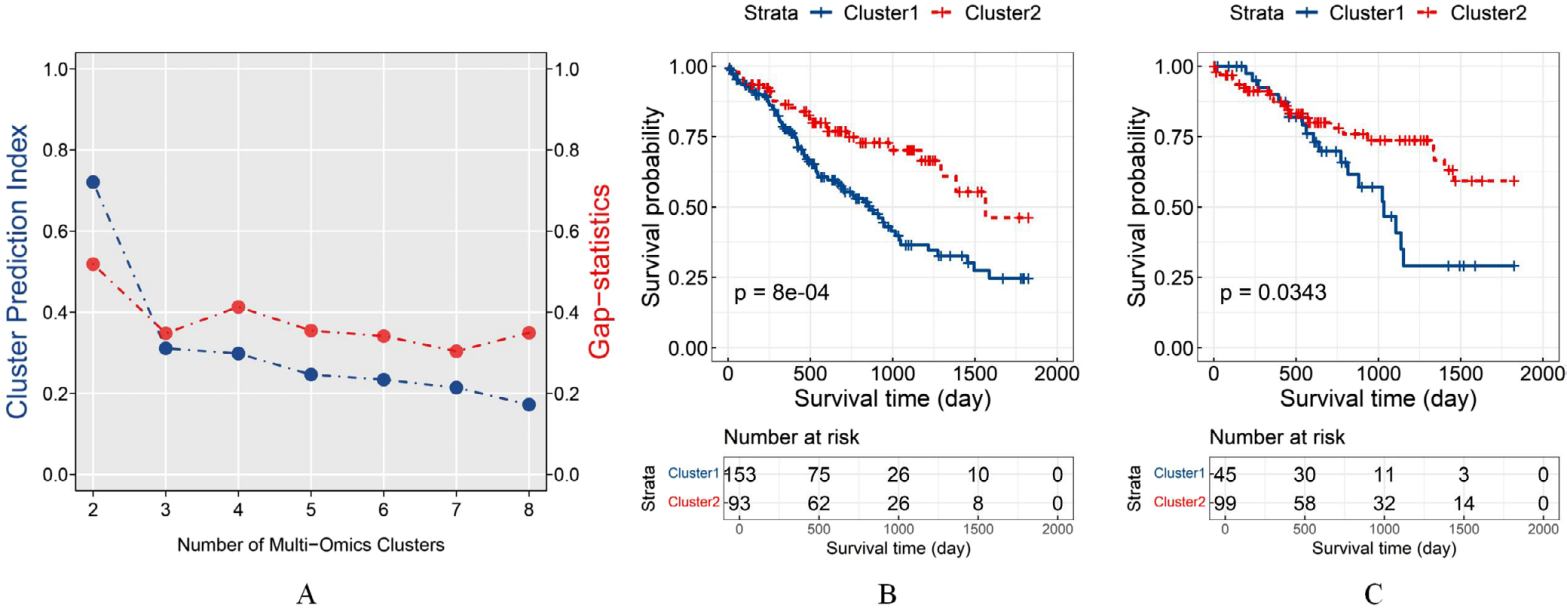
(A) Results of CPI and gap statistic for 2 to 8 clusters, choosing 2 as the optimal number of clusters. (B) Kaplan–Meier curves for the low‐ and high‐risk groups derived from the FHSXMU/SPPH dataset. (C) Kaplan–Meier survival curves for the low‐ and high‐risk groups derived from the TCGA/TCIA dataset. The statistical significance threshold was set at *p* < 0.05 for both datasets.

We chose the solution with *k* = 2 as optimal, using maximizing the sum of CPI and the gap statistic (Figure 2A). The results of the log‐rank test showed significant differences in the survival between the 2 subtypes (*χ* ^2^ = 11.2, *p* = 8E‐04) (Figure 2B). Therefore, Cluster 1 patients were defined as the high‐risk group, while Cluster 2 patients were defined as the low‐risk group.

### 3.2 Analysis of Phenotypic Subtypes in the FHSXMU/SPPH Dataset

The 246 glioma patients were divided into 2 phenotypic subtypes (153 patients in the high‐risk group and 93 patients in the low‐risk group) from the FHSXMU/SPPH dataset. Based on the two groups, we further examined the correlation between identified or hypothesized clinical and molecular risk factors for survival in glioma patients (Table 2A). There were no significant differences between the two groups in age or gender. The Chi‐square test suggested that there were significant differences in pathology grading (*χ* ^2^ = 22.098, *p* < 0.001), IDH genotype (*χ* ^2^ = 9.410, *p* = 0.002), and MGMT promoter genotype (*χ* ^2^ = 4.048, *p* = 0.044) between phenotypic subtypes.

**TABLE 2:** Baseline clinical and molecular data for different groups in both datasets.

Characteristics	High‐risk group	Low‐risk group	*χ* ^2^/*Z*	*p*
(A) FHSXMU/SPPH				
Age (year, *x* ± *s*)	48.71 ± 16.17	49.00 ± 13.29	0.656	0.512
Gender, *n* (%)			1.809	0.179
Female	79 (51.63)	57 (61.29)		
Male	74 (48.37)	36 (38.71)		
Grade, *n* (%)			22.098	**< 0.001**
2	36 (23.53)	46 (49.46)		
3	49 (32.03)	29 (31.18)		
4	68 (44.44)	18 (19.35)		
Tumor volume (cm^3^, *x* ± *s*)	54.24 ± 32.55	22.00 ± 27.09	9.260	**< 0.001**
IDH genotype, *n* (%)			9.410	**0.002**
Wild type	93 (60.78)	37 (39.78)		
Mutant type	60 (39.22)	56 (60.22)		
MGMT promoter, *n* (%)			4.048	**0.044**
Unmethylation	60 (39.22)	24 (25.81)		
Methylation	93 (60.78)	69 (74.19)		
(B) TCGA/TCIA				
Age (year, *x* ± *s*)	49.07 ± 13.24	46.84 ± 14.94	0.735	0.464
Gender, *n* (%)			0.129	0.719
Female	24 (53.33)	48 (48.48)		
Male	21 (46.67)	51 (51.52)		
Grade, *n* (%)			0.850	0.654
2	20 (44.44)	36 (36.36)		
3	19 (42.22)	48 (48.48)		
4	6 (13.33)	15 (15.15)		
Tumor volume (cm^3^, *x* ± *s*)	8.73 ± 9.75	58.63 ± 53.69	−7.900	**< 0.001**
IDH genotype, *n* (%)			4.520	**0.033**
Wild type	19 (42.22)	23 (23.23)		
Mutant type	26 (57.78)	76 (76.77)		
MGMT promoter, *n* (%)			1.914	0.167
Unmethylation	13 (28.89)	17 (17.17)		
Methylation	32 (71.11)	82 (82.83)		

*Note:* (A) FHSXMU/SPPH dataset. (B) TCGA/TCIA dataset. For continuous values, data are expressed as *mean* ± *standard* deviation (*x* ± *s*), and *p* values are derived from comparisons between the two clusters using the Mann–Whitney *U* test. For categorical values, data are frequency and composition ratio (*n*, %), and *p* values are derived from comparisons between the two clusters using the Chi‐Square test. The significance level for all analyses was set to 0.05. Statistically significant *p*‐values are highlighted in bold.

Controlling for age at initial diagnosis, gender, pathological grading, and tumor volume, we conducted Cox regression to explore the associations between the two subtypes and survival outcomes. The results are shown in Table 3. Patients in the high‐risk group were 1.759 times higher in risk of death than the low‐risk group (*p* = 0.020). The risk of mortality in patients older than 52 years was 2.881 times higher than in the low‐risk group (*p* < 0.001), and the risk of death of patients with CNS WHO grade 4 was 2.777 times higher than in patients with CNS WHO grade 2 (*p* < 0.001).

**TABLE 3:** Cox regression results of 246 glioma patients from FHSXMU/SPPH.

Variables	*b* (*S*. *E*)	*Z*	*p*	HR (95% CI)
Cluster				
High‐risk group*	0.565 (0.242)	2.334	**0.020**	1.759 (1.095, 2.827)
Age*(years, ≥ 52)	1.058 (0.225)	4.699	**< 0.001**	2.881 (1.853, 4.478)
Gender	0.034 (0.213)	0.159	0.873	1.035 (0.682, 1.570)
Grade				
3	0.500 (0.296)	1.692	0.091	1.649 (0.924, 2.943)
4*	1.022 (0.292)	3.504	**< 0.001**	2.777 (1.569, 4.918)
Tumor volume (cm^3^, ≥ 91)	−0.403 (0.346)	−1.166	0.244	0.668 (0.340, 1.316)

* *Note:* Shows statistical significance (*p* < 0.05); Here we take low‐risk subtype with better prognosis as the reference group for comparison, and take age < 52 years, tumor volume < 91 cm^3^, and WHO grade 2 as the reference grade for comparison. Statistically significant *p*‐values are highlighted in bold.

**FIGURE 3:**
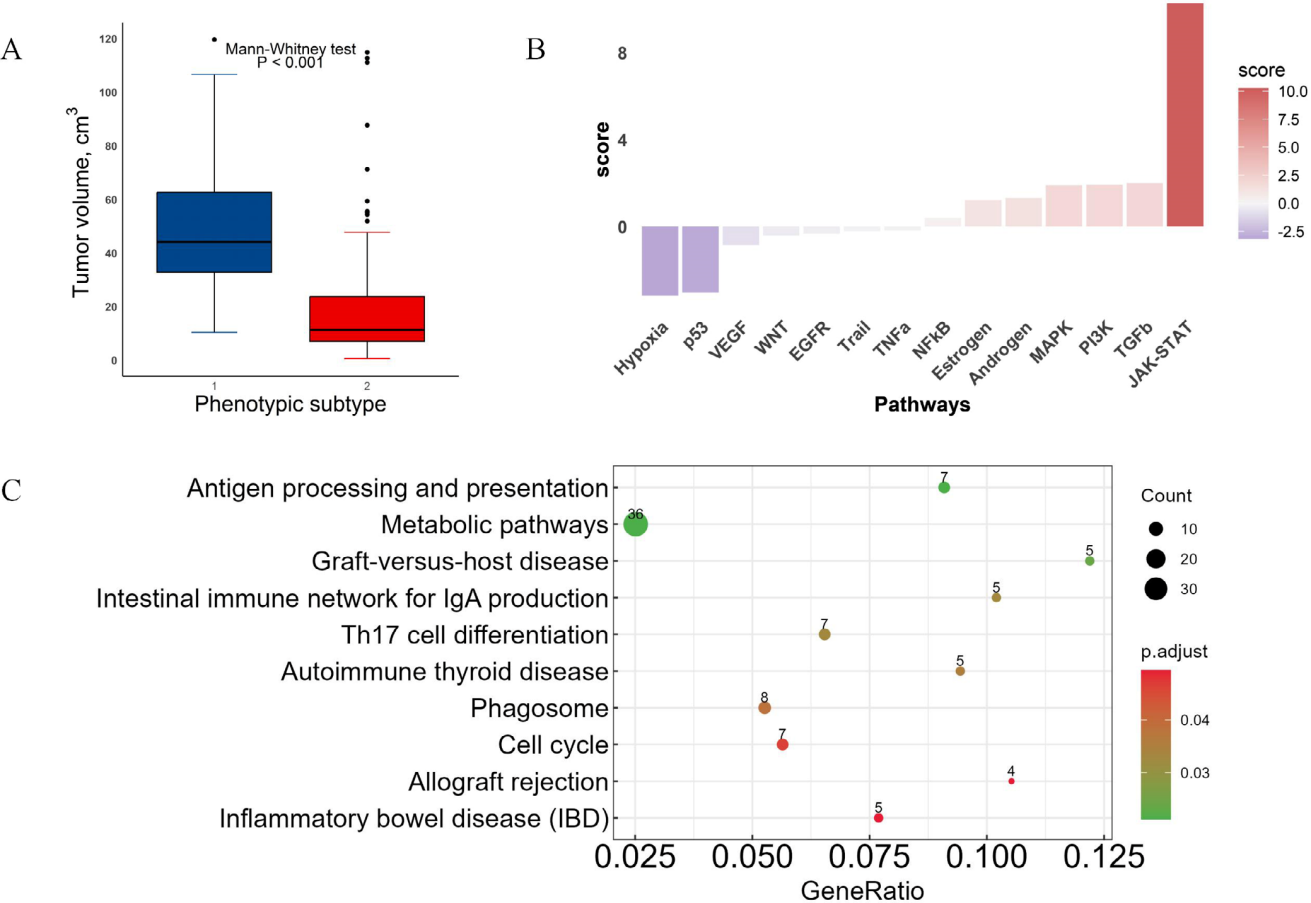
(A) Tumor volume by subtypes for the FHSXMU/SPPH dataset. The high‐risk group had significantly larger tumors compared to the low‐risk group (*p* < 0.001, Mann–Whitney test). There was no overlap in tumor volume between the high‐risk group and the low‐risk group. (B) Pathway analysis from TCGA/TCIA dataset. (C) KEGG enrichment analysis of genes.

Tumor volume was found to be significantly different between subtypes based on the Mann–Whitney *U*‐test (*p* < 0.001); smaller tumors were found in the low‐risk group, whereas larger tumors were in the high‐risk group (Figure 3A). Despite this correlation, tumor volume was not a sufficiently independent predictor of the phenotypic subtypes. The correlation coefficient between phenotypic subtypes and tumor volume was −0.456 (95% CI, −0.550 to −0.351). Moreover, in the Cox proportional hazards model, tumor volume was not a statistically significant predictor of phenotypic subtypes (*p* = 0.244).

### 3.3 Pathway and Enrichment Analysis of the Genes in the Key Modules

We predicted that 45 patients from the TCGA/TCIA dataset belonged to the high‐risk group, while 99 belonged to the low‐risk group. Survival analysis using the log‐rank test showed statistically significant results (*χ* ^2^ = 4.4, *p* = 0.0343), with Kaplan–Meier curves in Figure 2C. Relevant clinical data for each cluster are summarized in Table 2B.

Four signaling pathways showed significant differences between the two groups, JAK–STAT and TGF‐β were activated in the high‐risk group, while Hypoxia and p53 were activated in the low‐risk group (Table 4, Figure 3B). For the KEGG analysis, genes in the key modules were enriched in 10 pathways (BH FDR corrected *p*‐value < 0.05) (Figure 3C, Part 4 of Appendix S1).

**TABLE 4:** Pathway activity inference from DEG *t*‐values between the 2 groups.

	Pathway	*t*‐statistics	*p*
1	Androgen	1.312231	0.189457
2	EGFR	−0.3266	0.743974
3	Estrogen	1.212973	0.225154
4	Hypoxia	−3.17841	**0.001483**
5	JAK–STAT	10.26157	**1.21E‐24**
6	MAPK	1.897001	0.057842
7	NF‐κB	0.386127	0.699407
8	PI3K	1.919387	0.05495
9	TGF‐β	2.000366	**0.045474**
10	TNFα	−0.18352	0.854389
11	Trail	−0.20799	0.835236
12	VEGF	−0.85186	0.394302
13	WNT	−0.4005	0.688796
14	p53	−3.04011	**0.002368**

*Note:* The *t*‐statistic reflects the pathway score. If it is positive, we interpret the pathway as active; if negative, we interpret it as inhibited. The *t*‐statistic is considered meaningful only if *p* < 0.05. Statistically significant *p*‐values are highlighted in bold.

### 3.4 Model Construction and Performance Comparison

From the comparison of differences between subtypes, we found that there were significant differences in IDH genotype (*χ* ^2^ = 9.410, *P* = 0.002) between the two groups. We constructed predictive models for the IDH genotype in the FHSXMU/SPPH dataset.

In the FHSXMU/SPPH dataset, based on the stability feature selection procedure (penalized logistic regression) described previously, we obtained 99 reduced T1CE radiomic features and 27 reduced T2FLAIR radiomics features (Table S1). We compared the classification performance of five models, GA‐KPLS, RF, LASSO, KNN and NB, after 1000 iterations of resampling. Table 5 presents the predictive results for these models across various evaluation metrics. As shown in Table 5, all models except KNN achieved AUC values above 0.7 (ranging from 0.716 to 0.819). All models except RF had specificity (Sp) values greater than 0.7 (ranging from 0.744 to 0.847). Accuracy (ACC) values ranged from 0.694 for KNN to 0.742 for GA‐KPLS. Table 5 also highlights that the KNN model, while achieving a high specificity (Sp) of 0.847, has a lower sensitivity (Se) of 0.521. This suggests that the KNN model emphasizes accuracy in identifying the majority class.

**TABLE 5:** Model performance for predicting the IDH genotype.

Models	AUC	Se	Sp	ACC	Youden	F‐measure	MCC	G‐means
GA‐KPLS	**0.819**	0.740	0.744	**0.742**	**0.484**	**0.728**	**0.488**	**0.739**
RF	0.716	**0.774**	0.659	0.713	0.433	0.716	0.438	0.711
LASSO	0.721	0.693	0.750	0.723	0.443	0.700	0.448	0.717
KNN	0.684	0.521	**0.847**	0.694	0.368	0.610	0.394	0.660
NB	0.740	0.710	0.771	**0.742**	0.481	0.720	0.486	0.737

Abbreviations: ACC, accuracy; AUC, area under the curve; MCC, Matthews correlation coefficient; Se, sensitivity; Sp, specificity. The best performance across models for each metric is highlighted in bold.

**FIGURE 4:**
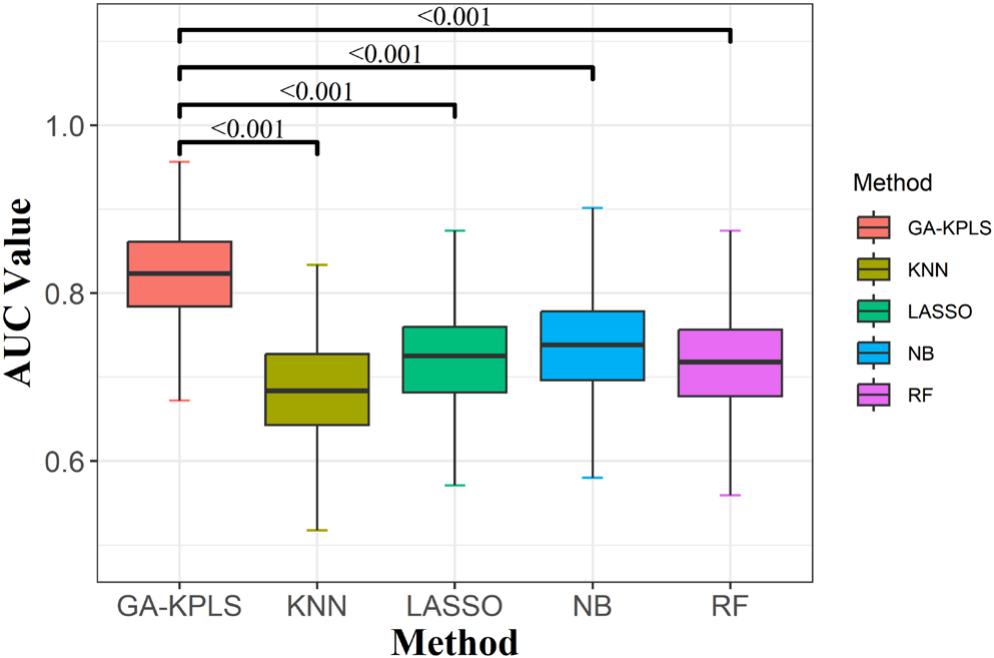
Boxplot of AUC values for the five different models predicting IDH genotype (based on 1000 random splits). The *y*‐axis represents the AUC value. *p* values were obtained using Dunnett's multiple‐comparison test.

The GA‐KPLS model exhibited strong performance across nearly all metrics, demonstrating its superior efficacy. To visualize the prediction results, we selected the AUC values as the composite evaluation metric, representing the results of 1000 random samples (Figure 4). The AUC value for the GA‐KPLS model is higher than those for RF, LASSO, KNN, and NB.

**FIGURE 5:**
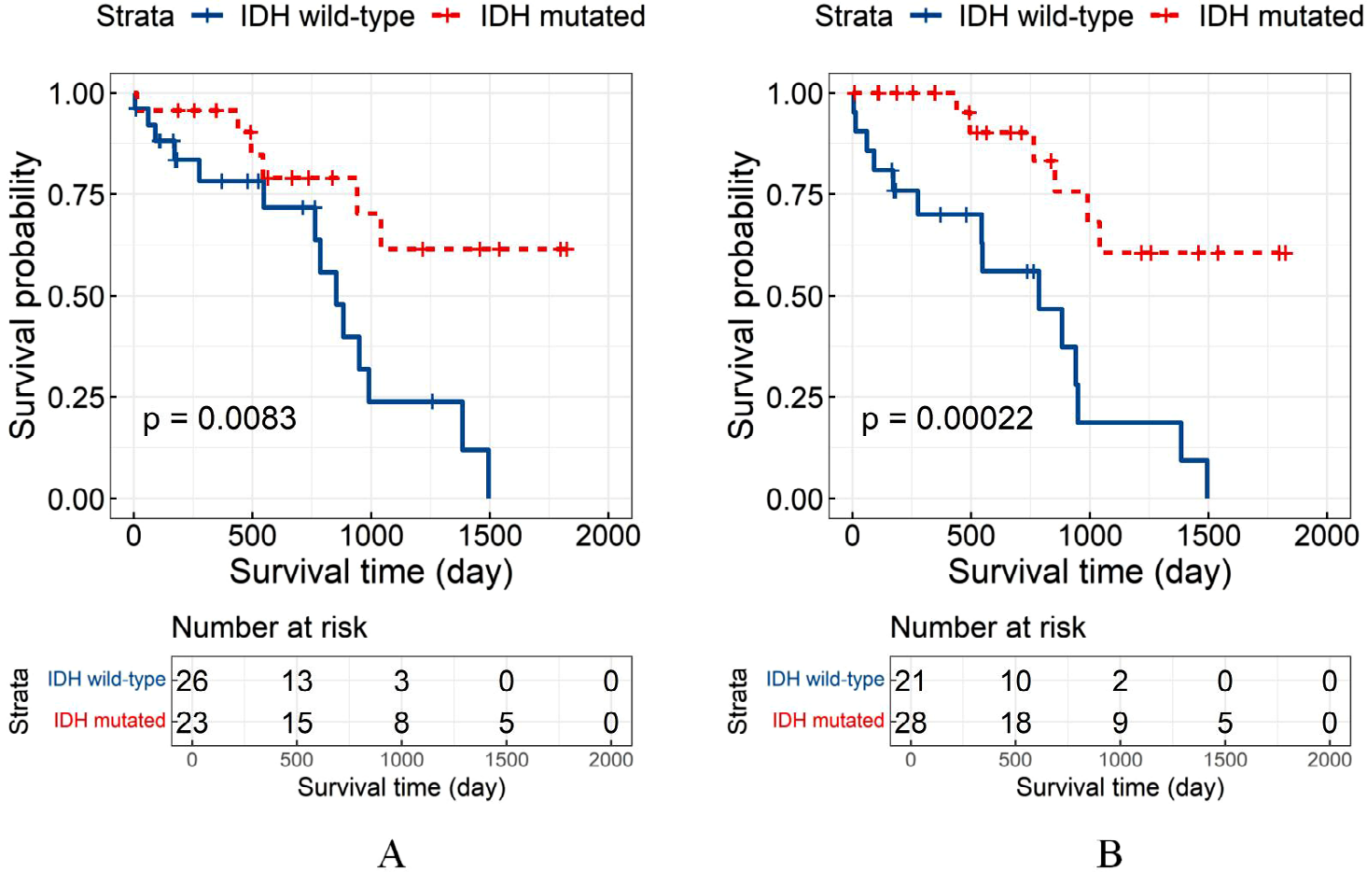
Kaplan–Meier survival curves comparing IDH‐mutated with nonmutated glioma patients. (A) The survival curve includes the original 49 patients in the testing cohort. (B) The survival curve is based on the predicted IDH genotype using the GA‐KPLS method. The statistical significance threshold was set at *p* < 0.05.

To demonstrate the clinical significance of identifying the IDH genotype, we selected 197 training and 49 testing samples from the FHSXMU/SPPH dataset, with a random split yielding an MCC = 0.484 (close to MCC_mean_ = 0.488). Kaplan–Meier curves based on the original and predicted data showed significantly different survival probabilities (*p* < 0.01). Figure 5A shows the original survival curve, and Figure 5B shows the survival curve based on new predictions with the GA‐KPLS method. These results indicate that GA‐KPLS performs well, as the survival curves for both original and predicted labels are similar. For predicting future events, the entire dataset can be used as a training set, and the obtained radiomic features can be applied to predict the IDH genotype of glioma patients.

### 3.5 Relative Contributions of Key Radiomic Features

**FIGURE 6:**
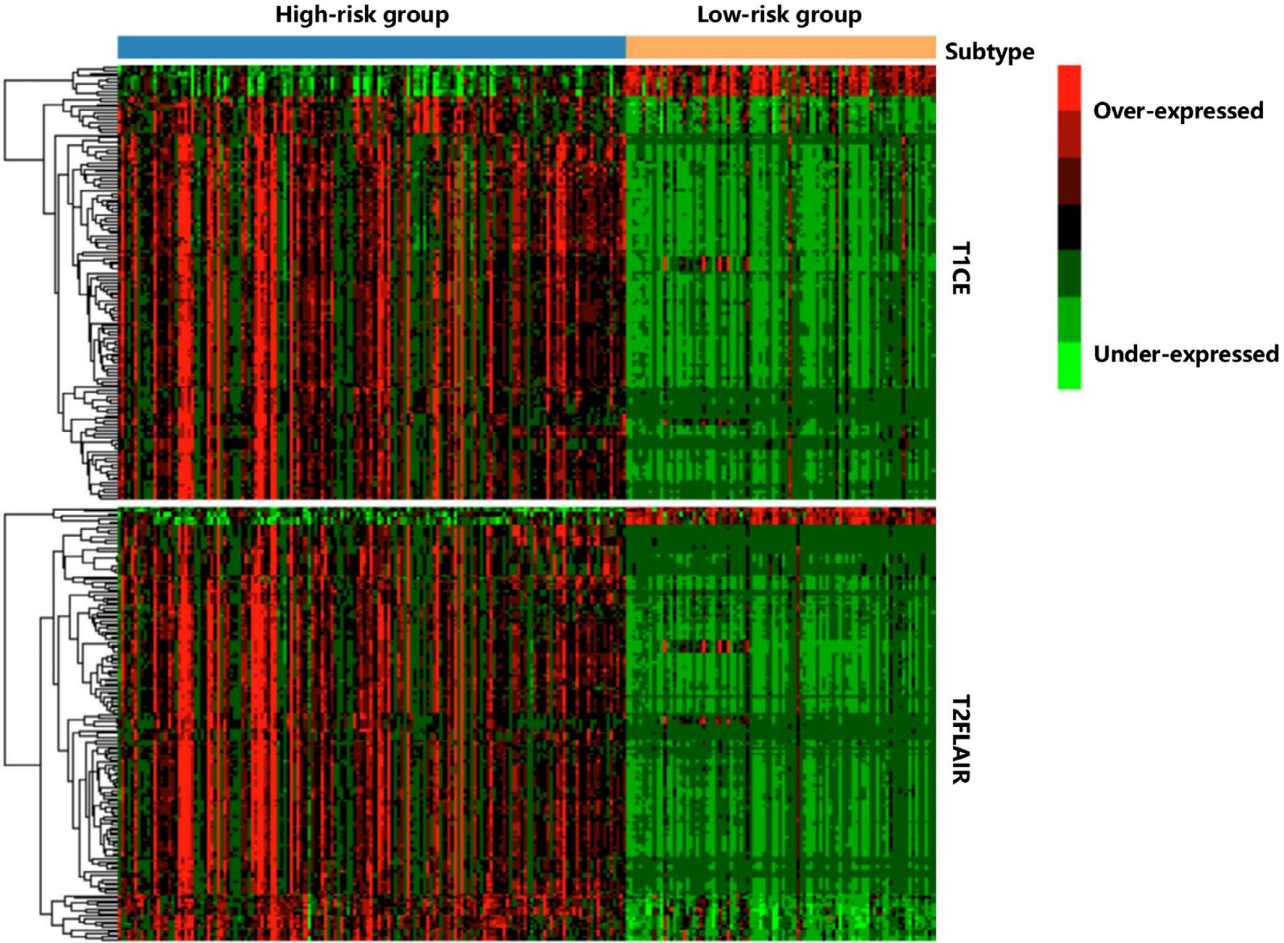
Heatmap of selected enriched radiomic features between the 2 subtypes. Each column represents a patient, and each row indicates an individual feature. Red and green color represents relatively over‐ and under‐expressed, respectively.

Based on the clusters identified by hMKL, we performed relative contributions of key imaging features between different clusters using the Kruskal‐Wallis *H*‐test and the hypergeometric distribution test. We set the FDR *q*‐value < 0.05 as the significance threshold. We identified a total of 141 T1CE features, among which 10 were over‐expressed and 131 were under‐expressed, and a total of 166 T2FLAIR features, of which 7 were overexpressed and 159 were under‐expressed. The heatmap in different sequence data is shown in Figure 6. These features are expressed differently between the two subtypes. The feature named Run Length Non‐Uniformity Normalized provides key insights into tumor heterogeneity, which is closely linked to malignancy, invasiveness, and poor prognosis. It reflects the tumor's non‐uniform structure and is highly relevant for assessing malignancy, heterogeneity, and prognosis [33].

## 4 Discussion

Gliomas are the most common primary malignant brain tumors [1], and despite available treatments such as surgery, radiotherapy, and chemotherapy, clinical outcomes remain suboptimal [34]. Accurate risk stratification before treatment is essential for developing individualized management plans.

In our study, we identified 4 signaling pathways showing statistical differences between the high‐ and low‐risk glioma subtypes. In the high‐risk group, activation of the JAK–STAT pathway promotes aberrant cell proliferation and resistance to apoptosis [35]. Concurrently, activation of the TGF‐β pathway further exacerbates tumor malignancy by inducing epithelial‐mesenchymal transition (EMT) and promoting tumor invasion, metastasis, and immune evasion [36, 37]. In contrast, the Hypoxia and p53 pathways were activated in the low‐risk group. The activation of the Hypoxia pathway may reflect an IDH mutation‐driven “pseudo‐hypoxic” state: the oncometabolite 2‐HG, produced by IDH mutations, inhibits prolyl hydroxylases, leading to the stabilization of HIF‐1α and activation of the Hypoxia pathway under normoxic conditions [38]. In low‐risk gliomas, this state may primarily promote angiogenesis to improve the local microenvironment or induce cells into a relatively quiescent state, thereby limiting malignant progression. Furthermore, the activation of the p53 pathway in the low‐risk group, as a classical tumor suppressor mechanism, inhibits tumor growth by promoting cell cycle arrest, DNA repair, and apoptosis [39]. Collectively, the activation of the Hypoxia and p53 pathways in the low‐risk group shapes its favorable prognostic features through microenvironmental adaptation and cell cycle regulation, respectively.

The feature Run Length Non‐Uniformity Normalized offers valuable insights into the heterogeneity of tumors, which is a critical factor associated with their malignancy, invasiveness, and overall prognosis [33]. This feature highlights the irregularities in the tumor's internal structure, indicating varying tissue characteristics that are often linked to more aggressive behavior. Its ability to capture structural non‐uniformity makes it a crucial metric for evaluating tumor malignancy, predicting clinical outcomes, and assessing the potential for metastasis, thereby providing important prognostic information.

IDH is a key rate‐limiting enzyme in the citric acid cycle, and mutations in this enzyme were first identified by Parsons et al. (2008) in GBM [40]. Their research demonstrated that glioma patients with IDH mutations who received concurrent chemoradiotherapy had significantly longer progression‐free survival compared to those who did not undergo such treatment [41]. Therefore, accurately assessing the IDH genotype in glioma patients holds substantial clinical value. Our study evaluated five predictive models and found that the GA‐KPLS model was particularly robust, achieving high accuracy in predicting the IDH genotype of glioma patients using radiomic features from T1CE and T2FLAIR sequences. By leveraging kernel functions, the GA‐KPLS model effectively captures nonlinear relationships between radiomic features and the IDH genotype, outperforming other predictive models. Unlike KNN, which relies on local similarities and ignores global data structure, GA‐KPLS employs global optimization to enhance prediction performance. Compared to NB, GA‐KPLS excels in comprehensive metrics such as AUC, Youden index, and MCC, achieving a better balance between sensitivity and specificity while minimizing misclassification risks. Although NB exhibits slightly higher specificity, its lower sensitivity may result in more false negatives. In contrast, the higher sensitivity of GA‐KPLS is clinically significant for IDH genotype prediction in gliomas, where accurate classification is critical for treatment selection and prognosis assessment. The superior AUC and balanced performance of GA‐KPLS contribute to improved prediction accuracy and reduced misdiagnosis risks, thus acknowledging its practical relevance in supporting clinicians to make more precise treatment decisions.

This study had several limitations. First, the analysis only included conventional sequences T1CE and T2FLAIR, without considering other potentially informative sequences such as apparent diffusion coefficient, diffusion‐weighted imaging, and diffusional kurtosis imaging. Expanding the dataset to include these could improve tumor characterization, but challenges like high computational demands and data standardization need to be addressed. Second, our study focused primarily on radiomic features, and potential interactions with clinical data should be explored in future research to better identify glioma subtypes. Third, differences in the distribution of IDH genotype between the training and test datasets may impact model performance and generalizability. Such imbalances could introduce bias in the prediction of certain subtypes and potentially confound other subtype‐specific findings. We should consider employing the SMOTE algorithm [42] to address potential class imbalance in future studies, which is designed to handle prediction with imbalanced data.

Our research demonstrates that high‐throughput image analysis can classify glioma subtypes (HGG and LGG) based on MRI, revealing biological differences to guide treatment strategies. We propose a GA‐KPLS‐based model for non‐invasive IDH mutation prediction, offering a surrogate for molecular tests. This approach aids preoperative decision‐making, providing real‐time insights into tumor dynamics and improving glioma diagnosis and treatment planning.

## Author Contributions

**Junyu Yan:** data curation (lead), investigation (lead), software (lead). **Min Hao:** data curation (equal), investigation (equal). **Tong Wang:** data curation (equal), validation (equal). **Qi Yang:** data curation (equal), software (equal), validation (equal). **Congcong Jia:** data curation (equal), visualization (equal). **Wenju Niu:** data curation (equal), investigation (equal). **Yan Tan:** resources (supporting), supervision (supporting). **Hui Zhang:** resources (supporting), supervision (supporting). **Hongyan Cao:** conceptualization (lead), methodology (equal), writing – review and editing (equal). **Guoqiang Yang:** methodology (equal), resources (supporting), writing – review and editing (equal).

## Funding

Our work was supported by the National Natural Science Foundation of China (U21A20386, 82473739, 82071893, 82371941), 10.13039/501100019447 ‐ Applied Basic Research Project of Shanxi Province (202303021211204, 202303021211130), Shanxi Province Research Funding Project for Returned Overseas Scholars (2024‐081), and Shanxi Province Higher Education “Billion Project” Science and Technology Guidance Project.

## Ethics Statement

This study at our institution was approved by the Ethics Committee of FHSXMU, with the approval number: 2021 K‐K073.

## Consent

Due to the retrospective nature of the study, the requirement for written informed consent was waived by the Institutional Review Board.

## Conflicts of Interest

The authors declare no conflicts of interest.

## Supporting information


**FIGURE S1:** A DBI results for 2 to 8 clusters, with 2 clusters selected as the optimal number. B CH index results for 2 to 8 clusters, with 2 clusters selected as the optimal number.
**Table S1:** Radiomic features derived from the stability feature selection.

## Data Availability

The data that support the findings of this study are available from the corresponding author upon reasonable request.
