# Supplementary material for: Identification of Glioma Phenotypic Subtypes From Multimodal MRI Data Using Hierarchical Multi‐Kernel Learning
Source: Cancer Med. 2026 Feb 1;15(2):e71572. doi: 10.1002/cam4.71572 (PMC12861564; doi:10.1002/cam4.71572)
Supplement: Supplementary file 1 — FIGURE S1: A DBI results for 2 to 8 clusters, with 2 clusters selected as the optimal number. B CH index results for 2 to 8 clusters, with 2 clusters selected as the optimal number. Table S1: Radiomic features derived from the stability feature selection. [file CAM4-15-e71572-s001.docx]

**Supplementary Materials for “Identification of glioma phenotypic subtypes from multimodal MRI data using hierarchical multi-kernel learning”**

Junyu Yan^1,2,3#^, Min Hao^1,4#^, Tong Wang^2,3#^, Qi Yang^3,5^, Congcong Jia^3,5^, Wenju Niu^1,4^, Yan Tan^1^, Hui Zhang^1,4,6,7^, Hongyan Cao^3,5*^, Guoqiang Yang^1,4,6*^

1 Department of Radiology, First Hospital of Shanxi Medical University, Taiyuan 030001, China

2 Academy of Medical Sciences, Shanxi Medical University, Taiyuan 030001, China

3 Department of Health Statistics, Shanxi Provincial Key Laboratory of Major Diseases Risk Assessment, School of Public Health, Shanxi Medical University, Taiyuan 030001, China

4 College of Medical Imaging, Shanxi Medical University, Taiyuan 030001, China

5 MOE Key Laboratory of Coal Environmental Pathogenicity and Prevention, Shanxi Medical University, Taiyuan 030001, China

6 Shanxi Key Laboratory of Intelligent Imaging and Nanomedicine, First Hospital of Shanxi Medical University, Taiyuan 030001, China

7 Intelligent Imaging Big Data and Functional Nano-imaging Engineering Research Center of Shanxi Province, First Hospital of Shanxi Medical University, Taiyuan 030001, China

^#^ These authors contributed equally to this work.

^*^ Correspondence:

Hongyan Cao, Department of Health Statistics, Shanxi Provincial Key Laboratory of Major Diseases Risk Assessment, School of Public Health, Shanxi Medical University, Taiyuan 030001, China.

Email: [caohy@sxmu.edu.cn](mailto:caohy@sxmu.edu.cn)

Guoqiang Yang, Department of Radiology, First Hospital of Shanxi Medical University, Taiyuan 030001, China

Email: [doctor_ygq@163.com](mailto:Doctor_ygq@163.com)

**Part 1. The specific process of hMKL**

Stage 1: Under the framework of Cancer Integration via Multikernel Learning (CIMLR)[1], the Gaussian kernel parameters and weights for each omics type are optimized. The form of the optimized objective function is defined as follows:

$$\min_{S,L,w} -\sum_{i,j,l} w_{l}K_{l}\left( x_{i},x_{j} \right)S_{ij}+\alpha\left\| S \right\|_{F}^{2}+\beta tr\left( L^{T}\left( I_{n}-S \right)L \right)+\rho\sum_{l} w_{l}logw_{l}$$

$subject to L^{T}L=I_{K}, \sum_{l} w_{l}=1, w_{l}\geq0, \sum_{j} S_{ij}=1, and S_{ij}\geq0$ (1)

where $\alpha$ and $\beta$ are two non-negative tuning parameters, $\left\| S \right\|_{F}$ denotes the Frobenius norm of the similarity matrix $S$, and $L$ is the low-dimensional auxiliary matrix used for the dimensionality reduction of matrix $S$. $I_{n}$ and $I_{C}$ represent the identity matrices of size $n\times n$ and $K\times K$, respectively, where $K$ is the predefined number of clusters.

Stage 2: Obtain the final weighted similarity matrix using unsupervised multiple kernel learning (UMKL)[2]. First, construct the connectivity matrix $W$ to reflect the topological structure of the original data, where $W_{ij}$ represents the number of connections between samples $\left( i,j \right)$ across multiple omics. Given the weight $\gamma$, introduce the N-dimensional vector $\Delta_{i}\left( \gamma\right)$ defined as $\Delta_{i}\left( \gamma\right)=\left( \begin{matrix} S_{i1}^{*} \\ \vdots\\ S_{in}^{*} \end{matrix} \right)$, where $S^{*}=\sum_{m=1}^{M} \gamma_{m}S^{m}$ denotes the linear combination of different fused kernels, and $S_{i1}^{*}\cdots S_{in}^{*}$ reflects the similarity between sample $i$ and others. Construct the objective function as follows:

$$\min_{\gamma} \sum_{i,j=1}^{n} W_{ij}\left\| \Delta_{i}\left( \gamma\right)-\Delta_{j}\left( \gamma\right) \right\|^{2}$$

$for S^{*}=\sum_{m=1}^{M} \gamma_{m}S^{m}$ (2)

$$\gamma\in\mathbb{R}^{M} such that \gamma_{m}\geq0 and \sum_{m=1}^{M} \gamma_{m}=1$$

where $S^{m}$ is the similarity matrix obtained from the CIMLR framework for the $m$th omics. Finally, optimize the objective function to obtain the weights $\gamma$, leading to the final fused matrix,

$S_{final}=\sum_{m=1}^{M} \gamma_{m}S^{mm'}, \gamma_{m}\geq0 and \sum_{m=1}^{M} \gamma_{m}=1$ (3)

where $\gamma_{m}$ represents the weights of the different similarity matrices, and $S^{mm'}$ denotes the similarity matrix obtained from the UMKL framework. Based on the final fusion kernel, K-means was employed to achieve unsupervised clustering.

**Part 2. The specific process of CPI and gap statistic**

The Clustering Prediction Index (CPI)[3] is a resampling-based cross-validation method used to estimate the optimal number of clusters $k1$. The data is divided into training and testing data, with the process repeated to generate multiple sample pairs. A clustering algorithm is trained on the training data to determine cluster centers, which are then used to assign test samples to the nearest cluster. The CPI is calculated as the average within-cluster sum of squares across all test samples. The CPI is calculated as follows:

$\text{CPI}(k)=\frac{1}{m}\sum_{i=1}^{m} \sum_{x\in X_{n_{2}\times p_{i}}^{i}} \left\| x-C_{i}^{\left( k1 \right)}(x) \right\|^{2}$ (4)

where $m$ represents the number of data divisions, $C_{i}^{\left( k1 \right)}(x)$ is the cluster center assigned to test sample *x*.

The gap statistic[4] provides a quantitative method to determine the optimal number of clusters $k2$ in a dataset by comparing the observed within-cluster sum of squares $W_{k2}$ with its expected value under a null reference distribution. The formula for maximizing the gap statistic is as follows:

$\text{Gap}_{n}(k)=E_{n}^{*}\left\{ \log\left( \sum_{r=1}^{k2} \frac{1}{2n_{r}}\sum_{i,i'\in C_{r}} d_{ii'} \right) \right\}-\log\left( \sum_{r=1}^{k2} \frac{1}{2n_{r}}\sum_{i,i'\in C_{r}} d_{ii'} \right)$ (5)

where $n$ is the sample size, $C_{r}$ represents each cluster, $i$ and $i'$ denote the observations within the cluster, and $d$ is the squared Euclidean distance.

**Part 3. Methodology of DBI and CH and Results of optimal number of clusters validation**

Davies-Bouldin Index (DBI)[5] measures the compactness and separation of clusters. It is defined as the similarity between each cluster and its most similar other cluster. The formula is as follows:

$DBI=\frac{1}{N}\sum_{i=1}^{N} \begin{aligned} \max\\ j\neq i \end{aligned}\left( \frac{S_{i}+S_{j}}{d\left( c_{i},c_{j} \right)} \right)$ (6)

where $S_{i}$ and $S_{j}$ are the intra-cluster distances (average distance of points in cluster $i$ and $j$ to their centroids), and $d\left( c_{i},c_{j} \right)$ is the distance between the centroids of cluster $i$ and $j$. A higher similarity indicates poor separation between the clusters, while a lower similarity indicates better separation.

For each cluster $i$, DBI selects the most similar cluster $j$, and the final DBI is the average of the worst similarity scores. A lower DBI value indicates tighter and better-separated clusters. From Figure R1A, we can conclude that the optimal number of clusters is 2.

The essence of the Calinski-Harabasz index (CH)[6] is the ratio of the between-cluster distance to the within-cluster distance, and its formula is as follows:

$CH=\frac{\sum_{j=1}^{k} n_{j}||u_{j}-u||^{2}/(k-1)}{\sum_{j=1}^{k} \sum_{i=1}^{n_{j}} ||x_{i}-u_{j}||^{2}/(n-k)}$ (7)

where $k$ represents the total number of clusters, $u$ denotes the global mean, $u_{j}$ represents the mean of the $j$-th cluster, $n$ is the total number of samples, and $n_{j}$ represents the number of samples in the $j$-th cluster. A higher value of this function indicates better clustering performance. From Figure R1B, we can conclude that the optimal number of clusters is 2.


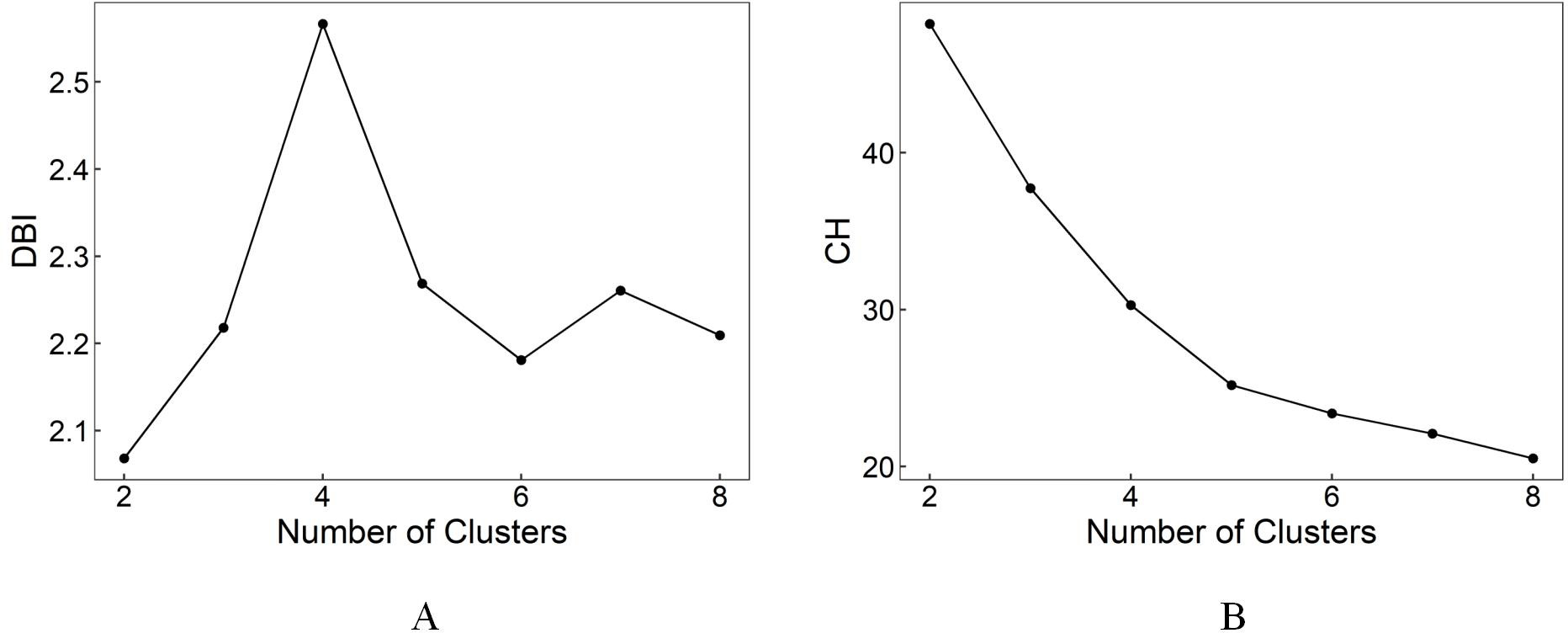


**Figure S1. A** DBI results for 2 to 8 clusters, with 2 clusters selected as the optimal number. **B** CH index results for 2 to 8 clusters, with 2 clusters selected as the optimal number.

**Part 4. Gene functional enrichment analysis**

We conducted KEGG pathway enrichment analysis to gain insight into the functional characteristics of the genes. The analysis revealed that a total of 10 KEGG pathways were enriched, as shown in **Fig. 3C**. In KEGG pathway enrichment analysis, the hub genes were significantly enriched in multiple disease-related signaling pathways, including Antigen processing and presentation, Metabolic pathways, and Th17 cell differentiation. Antigen processing and presentation plays a crucial role in the immune system’s recognition and clearance of tumor cells, and its dysregulation may lead to immune escape and tumor progression[7]. Samad noted that, compared to normal cells, cancer cells adopt altered metabolic pathways. Therefore, metabolic dysregulation in normal cells can be considered a fundamental hallmark of cancer, providing a theoretical basis for the clinical identification of cancer[8]. Th17 cell differentiation is critical in inflammation and autoimmune diseases, and its abnormal activation may be related to the immunosuppressive state of the tumor microenvironment[9].

**Part 5. Supplementary table**

**Table S1. Radiomic features derived from the stability feature selection**

| **T1CE** | |
| --- | --- |
| 1 | T1CE_wavelet.LLL_glszm_SmallAreaHighGrayLevelEmphasis |
| 2 | T1CE_wavelet.HHL_glszm_GrayLevelVariance |
| 3 | T1CE_wavelet.HLL_glszm_SizeZoneNonUniformityNormalized |
| 4 | T1CE_wavelet.LHL_glszm_GrayLevelNonUniformityNormalized |
| 5 | T1CE_gradient_firstorder_Minimum |
| 6 | T1CE_wavelet.LLH_glrlm_ShortRunHighGrayLevelEmphasis |
| 7 | T1CE_square_firstorder_Minimum |
| 8 | T1CE_squareroot_glszm_SizeZoneNonUniformity |
| 9 | T1CE_wavelet.LHL_glszm_GrayLevelNonUniformity |
| 10 | T1CE_wavelet.HHL_glrlm_LongRunLowGrayLevelEmphasis |
| 11 | T1CE_wavelet.LHH_firstorder_Maximum |
| 12 | T1CE_wavelet.LHL_firstorder_Range |
| 13 | T1CE_log.sigma.1.mm.3D_glszm_LargeAreaHighGrayLevelEmphasis |
| 14 | T1CE_lbp.3D.k_glrlm_LongRunHighGrayLevelEmphasis |
| 15 | T1CE_wavelet.LHL_firstorder_Variance |
| 16 | T1CE_wavelet.LLH_glrlm_LowGrayLevelRunEmphasis |
| 17 | T1CE_logarithm_glszm_GrayLevelNonUniformityNormalized |
| 18 | T1CE_gradient_glszm_GrayLevelNonUniformityNormalized |
| 19 | T1CE_lbp.3D.k_glcm_ClusterProminence |
| 20 | T1CE_gradient_firstorder_Variance |
| 21 | T1CE_lbp.3D.m1_gldm_SmallDependenceHighGrayLevelEmphasis |
| 22 | T1CE_square_gldm_DependenceNonUniformityNormalized |
| 23 | T1CE_exponential_glszm_LowGrayLevelZoneEmphasis |
| 24 | T1CE_wavelet.LLH_glszm_LowGrayLevelZoneEmphasis |
| 25 | T1CE_original_glszm_SizeZoneNonUniformity |
| 26 | T1CE_wavelet.LHH_glrlm_LowGrayLevelRunEmphasis |
| 27 | T1CE_exponential_gldm_HighGrayLevelEmphasis |
| 28 | T1CE_wavelet.LHL_gldm_LowGrayLevelEmphasis |
| 29 | T1CE_wavelet.LHL_gldm_DependenceNonUniformityNormalized |
| 30 | T1CE_wavelet.LLL_firstorder_Kurtosis |
| 31 | T1CE_wavelet.HLH_glszm_LowGrayLevelZoneEmphasis |
| 32 | T1CE_wavelet.LLL_gldm_LargeDependenceLowGrayLevelEmphasis |
| 33 | T1CE_gradient_glcm_ClusterProminence |
| 34 | T1CE_wavelet.HLL_gldm_DependenceVariance |
| 35 | T1CE_gradient_firstorder_MeanAbsoluteDeviation |
| 36 | T1CE_wavelet.LHL_glszm_SmallAreaLowGrayLevelEmphasis |
| 37 | T1CE_wavelet.HHH_glcm_MaximumProbability |
| 38 | T1CE_wavelet.LHL_firstorder_RootMeanSquared |
| 39 | T1CE_original_shape_MajorAxisLength |
| 40 | T1CE_exponential_glcm_Correlation |
| 41 | T1CE_lbp.3D.k_glcm_ClusterShade |
| 42 | T1CE_log.sigma.1.mm.3D_glszm_SizeZoneNonUniformity |
| 43 | T1CE_wavelet.LLH_firstorder_Kurtosis |
| 44 | T1CE_wavelet.LLH_gldm_LargeDependenceLowGrayLevelEmphasis |
| 45 | T1CE_lbp.3D.m2_gldm_LargeDependenceLowGrayLevelEmphasis |
| 46 | T1CE_exponential_firstorder_RobustMeanAbsoluteDeviation |
| 47 | T1CE_logarithm_glszm_SizeZoneNonUniformity |
| 48 | T1CE_lbp.3D.m2_ngtdm_Strength |
| 49 | T1CE_lbp.3D.m1_firstorder_TotalEnergy |
| 50 | T1CE_squareroot_firstorder_Variance |
| 51 | T1CE_wavelet.HHL_glszm_LowGrayLevelZoneEmphasis |
| 52 | T1CE_exponential_firstorder_InterquartileRange |
| 53 | T1CE_wavelet.LHL_glszm_LowGrayLevelZoneEmphasis |
| 54 | T1CE_wavelet.HLH_glcm_ClusterShade |
| 55 | T1CE_wavelet.HHL_glrlm_LowGrayLevelRunEmphasis |
| 56 | T1CE_wavelet.HHL_gldm_LargeDependenceLowGrayLevelEmphasis |
| 57 | T1CE_wavelet.HHH_gldm_DependenceNonUniformityNormalized |
| 58 | T1CE_wavelet.HLH_glrlm_ShortRunLowGrayLevelEmphasis |
| 59 | T1CE_wavelet.HLL_ngtdm_Complexity |
| 60 | T1CE_wavelet.LLL_gldm_SmallDependenceLowGrayLevelEmphasis |
| 61 | T1CE_logarithm_glcm_Correlation |
| 62 | T1CE_exponential_glszm_HighGrayLevelZoneEmphasis |
| 63 | T1CE_gradient_firstorder_RobustMeanAbsoluteDeviation |
| 64 | T1CE_exponential_glrlm_LongRunHighGrayLevelEmphasis |
| 65 | T1CE_wavelet.LLH_ngtdm_Strength |
| 66 | T1CE_wavelet.LHH_firstorder_Energy |
| 67 | T1CE_wavelet.LLH_glszm_SizeZoneNonUniformity |
| 68 | T1CE_wavelet.HLH_glszm_GrayLevelNonUniformityNormalized |
| 69 | T1CE_wavelet.LLL_glrlm_LongRunHighGrayLevelEmphasis |
| 70 | T1CE_wavelet.HLL_glszm_LowGrayLevelZoneEmphasis |
| 71 | T1CE_wavelet.LHL_glrlm_LowGrayLevelRunEmphasis |
| 72 | T1CE_wavelet.LHH_firstorder_Kurtosis |
| 73 | T1CE_wavelet.LHL_glcm_Correlation |
| 74 | T1CE_wavelet.LHL_glrlm_LongRunLowGrayLevelEmphasis |
| 75 | T1CE_wavelet.HHL_glrlm_RunVariance |
| 76 | T1CE_lbp.3D.k_glszm_GrayLevelVariance |
| 77 | T1CE_wavelet.LLH_glrlm_LongRunLowGrayLevelEmphasis |
| 78 | T1CE_exponential_glszm_SizeZoneNonUniformityNormalized |
| 79 | T1CE_wavelet.HLH_glcm_ClusterProminence |
| 80 | T1CE_lbp.3D.k_glcm_ClusterTendency |
| 81 | T1CE_original_shape_Maximum3DDiameter |
| 82 | T1CE_wavelet.LHH_glszm_LowGrayLevelZoneEmphasis |
| 83 | T1CE_wavelet.HHL_glszm_GrayLevelNonUniformityNormalized |
| 84 | T1CE_wavelet.HHH_glszm_LowGrayLevelZoneEmphasis |
| 85 | T1CE_square_firstorder_RootMeanSquared |
| 86 | T1CE_exponential_gldm_DependenceNonUniformityNormalized |
| 87 | T1CE_wavelet.HHH_gldm_DependenceVariance |
| 88 | T1CE_logarithm_glszm_SmallAreaLowGrayLevelEmphasis |
| 89 | T1CE_lbp.3D.k_glrlm_RunVariance |
| 90 | T1CE_wavelet.LHL_firstorder_Kurtosis |
| 91 | T1CE_square_glrlm_LongRunHighGrayLevelEmphasis |
| 92 | T1CE_square_glszm_LargeAreaHighGrayLevelEmphasis |
| 93 | T1CE_original_glcm_Correlation |
| 94 | T1CE_squareroot_glcm_MaximumProbability |
| 95 | T1CE_original_ngtdm_Complexity |
| 96 | T1CE_log.sigma.1.mm.3D_firstorder_90Percentile |
| 97 | T1CE_logarithm_firstorder_Maximum |
| 98 | T1CE_exponential_glszm_SmallAreaLowGrayLevelEmphasis |
| 99 | T1CE_lbp.3D.m1_gldm_LargeDependenceLowGrayLevelEmphasis |
| **FLAIR** | |
| 1 | FLAIR_wavelet.LHL_glcm_DifferenceVariance |
| 2 | FLAIR_logarithm_firstorder_Minimum |
| 3 | FLAIR_gradient_glcm_DifferenceVariance |
| 4 | FLAIR_wavelet.LLH_glcm_DifferenceVariance |
| 5 | FLAIR_wavelet.LLH_firstorder_Maximum |
| 6 | FLAIR_lbp.3D.m1_gldm_DependenceVariance |
| 7 | FLAIR_wavelet.LLH_glrlm_ShortRunLowGrayLevelEmphasis |
| 8 | FLAIR_gradient_glcm_Correlation |
| 9 | FLAIR_original_shape_Maximum2DDiameterRow |
| 10 | FLAIR_logarithm_glszm_GrayLevelNonUniformityNormalized |
| 11 | FLAIR_log.sigma.1.mm.3D_glrlm_ShortRunLowGrayLevelEmphasis |
| 12 | FLAIR_wavelet.LLH_glcm_ClusterProminence |
| 13 | FLAIR_wavelet.LHL_glrlm_ShortRunHighGrayLevelEmphasis |
| 14 | FLAIR_logarithm_glszm_HighGrayLevelZoneEmphasis |
| 15 | FLAIR_log.sigma.1.mm.3D_glcm_ClusterProminence |
| 16 | FLAIR_logarithm_glszm_LargeAreaLowGrayLevelEmphasis |
| 17 | FLAIR_wavelet.HLL_firstorder_TotalEnergy |
| 18 | FLAIR_wavelet.LLH_gldm_SmallDependenceLowGrayLevelEmphasis |
| 19 | FLAIR_exponential_glszm_LargeAreaLowGrayLevelEmphasis |
| 20 | FLAIR_wavelet.LLH_firstorder_TotalEnergy |
| 21 | FLAIR_wavelet.LHL_glszm_GrayLevelNonUniformity |
| 22 | FLAIR_log.sigma.1.mm.3D_glcm_DifferenceVariance |
| 23 | FLAIR_log.sigma.1.mm.3D_firstorder_Kurtosis |
| 24 | FLAIR_wavelet.LHH_firstorder_RobustMeanAbsoluteDeviation |
| 25 | FLAIR_logarithm_glszm_SizeZoneNonUniformity |
| 26 | FLAIR_wavelet.LHH_gldm_LargeDependenceLowGrayLevelEmphasis |
| 27 | FLAIR_wavelet.LLL_glrlm_LongRunLowGrayLevelEmphasis |

**References**

1. Ramazzotti D, Lal A, Wang B, et al. Multi-omic tumor data reveal diversity of molecular mechanisms that correlate with survival[J]. Nature communications, 2018, 9(1): 1-14.
2. Mariette J, Villa-Vialaneix N. Unsupervised multiple kernel learning for heterogeneous data integration[J]. Bioinformatics, 2018, 34(6): 1009-1015.
3. Chalise P, Fridley B L. Integrative clustering of multi-level ‘omic data based on non-negative matrix factorization algorithm[J]. PloS one, 2017, 12(5): e0176278.
4. Tibshirani R, Walther G, Hastie T. Estimating the number of clusters in a data set via the gap statistic[J]. Journal of the Royal Statistical Society: Series B (Statistical Methodology), 2001, 63(2): 411-423.
5. Davies D L, Bouldin D W. A cluster separation measure[J]. IEEE transactions on pattern analysis and machine intelligence, 1979 (2): 224-227.
6. Caliński T, Harabasz J. A dendrite method for cluster analysis[J]. Communications in Statistics-theory and Methods, 1974, 3(1): 1-27.
7. Pishesha N, Harmand T J, Ploegh H L. A guide to antigen processing and presentation[J]. Nature Reviews Immunology, 2022, 22(12): 751-764.
8. Samad A, Samant R, Rao K V, et al. Oxaloacetate as a Holy Grail Adjunctive Treatment in Gliomas: A Revisit to Metabolic Pathway[J]. Cureus, 2023, 15(11).
9. Hirahara K, Ghoreschi K, Laurence A, et al. Signal transduction pathways and transcriptional regulation in Th17 cell differentiation[J]. Cytokine & growth factor reviews, 2010, 21(6): 425-434.
